# Unfamiliar face matching ability predicts the slope of face learning

**DOI:** 10.1038/s41598-023-32244-w

**Published:** 2023-03-31

**Authors:** Kristen A. Baker, Catherine J. Mondloch

**Affiliations:** Department of Psychology, Brock University, 1812 Sir Isaac Brock Way, St. Catharines, ON L2S 3A1 Canada

**Keywords:** Human behaviour, Psychology, Learning and memory

## Abstract

We provide the first examination of individual differences in the efficiency of face learning. Investigating individual differences in face learning can illuminate potential mechanisms and provide greater understanding of why certain individuals might be more efficient face learners. Participants completed two unfamiliar face matching tasks and a learning task in which learning was assessed after viewing 1, 3, 6, and 9 images of to-be-learned identities. Individual differences in the slope of face learning (i.e., increases in sensitivity to identity) were predicted by the ability to discriminate between matched (same-identity) vs. mismatched (different-identity) pairs of wholly unfamiliar faces. A Dual Process Signal Detection model showed that three parameters increased with learning: Familiarity (an unconscious type of memory that varies in strength), recollection-old (conscious recognition of a learned identity), and recollection-new (conscious/confident rejection of novel identities). Good (vs. poor) matchers had higher Recollection-Old scores throughout learning and showed a steeper increase in Recollection-New. We conclude that good matchers are better able to capitalize on exposure to within-person variability in appearance, an effect that is attributable to their conscious memory for both learned and novel faces. These results have applied implications and will inform contemporary and traditional models of face identification.

## Introduction

Adults have a remarkable ability to recognize familiar faces despite within-person variability in appearance (e.g., changes in appearance resulting from lighting, hairstyle, expression, viewpoint, health). Familiar faces are recognized even when image quality is poor^1,2^, faces are far away^3^, disguised^4^, or are of a different race than the perceiver^5^. In contrast, matching identity of unfamiliar faces is error prone, even when viewing high-quality photos of upright own-race faces taken just moments apart^6–11^. Poor performance when viewing unfamiliar faces reflects the inherent challenge of face identification: Images of different faces can be very similar, and different images of the same face can vary in appearance.

The stark difference between recognition of unfamiliar vs. familiar faces, combined with the fact that every familiar face was once unfamiliar, raises a critical question: How does a newly encountered face become familiar? Exposure to within-person variability in appearance is key^12–18^. Viewing multiple high-variability images of a target identity (i.e., ambient images taken on different days) improves performance in lineup tasks^19,20^, same/different tasks, name verification tasks^21^, and old/new face identification tasks in which test stimuli comprise novel images of a learned identity^13,17^. Semantic associations also play a role. Recognition improves after social (*how trustworthy is this person?*) relative to perceptual (*how round is this face?*) judgements—a difference associated with increased neural activity in social processing regions (e.g., dorsal medial prefrontal cortex)^22^. Neural signatures of face learning have also been identified. The N250, an event-related potential component that reflects recognition, is more negative for new images of learned identities than for images of wholly unfamiliar identities in an implicit learning paradigm^12^, and for personally familiar vs. celebrity faces^23^. The Sustained Familiarity Effect, an event related potential occurring at approximately 400–600 ms, tracks level of familiarity with an identity^24^.

In the current study we provide the first examination of individual differences in the efficiency with which a newly learned face becomes familiar (i.e., the slope of face learning). The individual differences approach can provide valuable insights about the perceptual and cognitive mechanisms underlying face identification but is a largely untapped resource^25^. Individual differences in unfamiliar face identification are reliable^25–27^ and consistent across tasks that vary in the type of judgment being made (e.g., same/different vs. detecting a target in a lineup), the amount of within-person variability in appearance, and sequential versus simultaneous stimulus presentation^26^. To date, no study has examined whether individual differences in unfamiliar face matching predict improvement in performance as additional images are presented (i.e., the ability to capitalize on variability as a face becomes familiar).

The primary purpose of our study was to test the hypothesis that individual differences in unfamiliar face matching predict individual differences in the slope of face learning, as measured by d′ (sensitivity to identity). We predicted that individuals who are better at matching identity in wholly unfamiliar faces would have a steeper face learning slope based on several findings. First, group differences in unfamiliar face identification correspond to group differences in face learning. Adults from small towns perform worse when sorting images of unfamiliar faces than adults from large cities^28^; children perform worse on unfamiliar face identification tasks than adults^29^; and adults perform worse when tested with inverted or other-race faces as compared to upright or own-race faces^5,30^. These same group differences are seen in face learning and memory tasks^13,32,33^. Furthermore, individual differences on tasks measuring face memory (e.g., Models of Memory Test^27^, Cambridge Face Memory Test [CFMT]^34^) correlate with individual differences on unfamiliar face matching tests^27,35–37^. Such tasks measure performance at only one point during face learning; we examined performance as learning unfolded.

The second purpose was to examine whether the contribution of two processes known to underlie learning/memory (recollection and familiarity) differs between good vs. poor matchers while learning unfolds. To address this question, we analyzed our data using the Dual Process Signal Detection model (DPSD).

### Dual process signal detection

Two distinct processes (recollection and familiarity) influence recall of an event: Explicit memory details (recollection) and an implicit sense of familiarity^38–41^. The DPSD model proposes that recognition is influenced by three parameters: Recollection old (Ro), recollection new (Rn), and familiarity (Fam). The Ro parameter is a threshold process reflecting high-confidence hits. It is an episodic process in which high-quality information about the recollected stimulus is retrieved (e.g., *She played Black Widow in the Avengers movie*). The Rn parameter is a threshold process reflecting high-confidence correct rejections, sometimes considered to be more perceptual in nature^42^. The Fam parameter is a graded signal process that occurs at all levels of confidence, but in the absence of conscious recollection (e.g., *I think I have seen her before but can’t remember where*)^38–41^.

Previous findings suggest that individual differences in the slope of face learning might be associated with individual differences in recollection and/or familiarity. First, when asked to recognize actors from a familiar TV series, more inaccurate “familiar” responses and less recall of accurate semantic detail about a target (akin to recollection) is associated with poor performance on the CFMT^43^. Second, groups that perform poorly on face recognition tasks (e.g., older adults, adults tested with other-race faces) make fewer recollection-based responses than better performing groups (i.e., young adults, adults tested with own-race faces)^43–49^. To examine this question directly, we asked participants in the face learning task to report their confidence on a scale from 1 (certain that the image belongs to the target) to 6 (certain that the image does not belong to the target); binary responses (*same/different*) are not ideal for distinguishing responses based on recollection vs. familiarity.

### The current study

**Figure 1:**
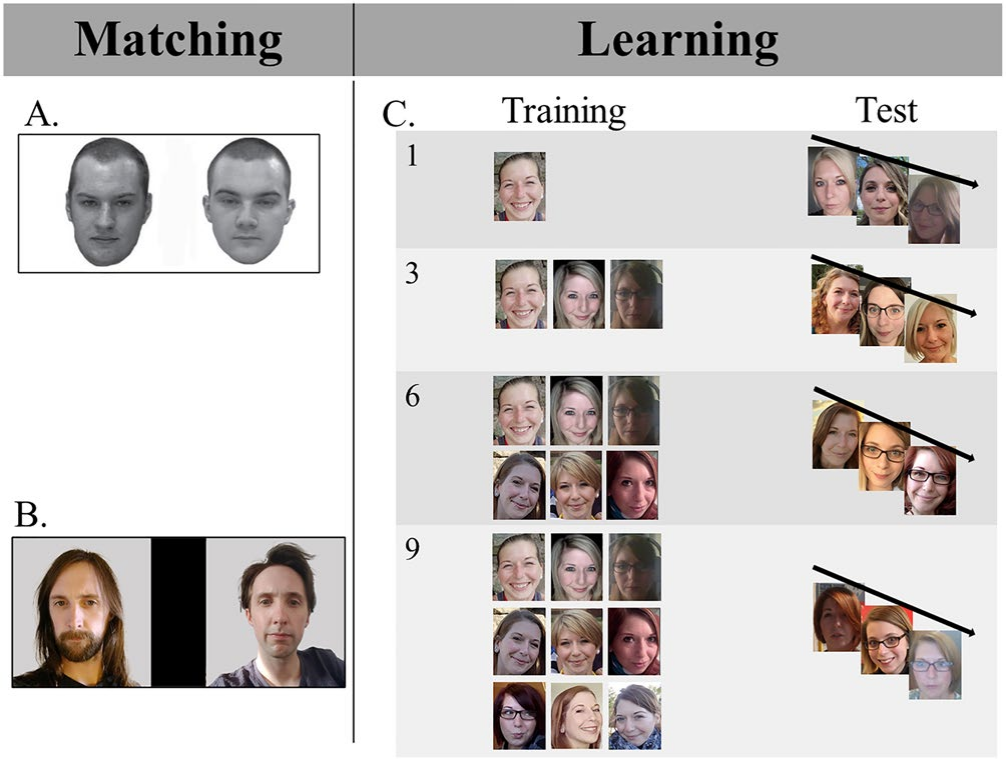
A depiction of the GFMT (**A**), AIFMT (**B**), and learning task (**C**). Participants first completed the GFMT (*n*_trials_ = 40; 50% match). They were instructed to press the “f” key if both images showed the same person and the “j” key if the images showed different people. Next, participants completed the AIFMT (*n*_trials_ = 80; 50% match); participants indicated their responses on a scale from 1 (certain that the images belong to the same person) to 6 (certain that the images belong to different individuals). Selecting 1, 2 or 3 was considered a match response and selecting 4, 5 or 6 was considered a mismatch response. Finally, participants completed the face learning task. Participants viewed 1, 3, 6, and then 9 images of a target identity. Their ability to recognize novel images of the learned identity, when intermixed with images of a similar distractor, was assessed after each of the learning phases. Selecting 1, 2 or 3 was considered an old response and selecting 4, 5 or 6 was considered a new response. Participants completed this learning procedure four times, once for each of 4 learning identities (*n*_test trials_ = 40). Images in (**B**) and (**C**) are for illustrative purposes and are photos of individuals not used in the experiment. We obtained informed consent for open-access publication from these models. The image in (**A**) was used in a prior publication^11^; permission to use this figure was granted.

Participants completed a battery of three face identification tasks. Two tasks (The Glasgow Face Matching Test [GFMT]^11^; The Ambient Image Face Matching Task [AIFMT]^26,50^) measured face matching in wholly unfamiliar faces. We developed a novel face learning task to measure the slope of face learning as participants were exposed to an increasing number of new images (1, 3, 6, and then 9 images) of a previously unfamiliar face. See Fig. 1 for a depiction of the tasks.

Our primary goal was to provide the first examination of the extent to which individual differences in face-matching abilities (using average d′) predict the slope of face learning (using d′ across four phases [1, 3, 6, and 9 images] in the learning task). We examined whether face matching ability (e.g., poor, mid-level, and good) interacted with learning phase such that good matchers benefitted more from the additional variability provided at each step of learning. We predicted that there would be a significant interaction, such that poor matchers would benefit less from viewing additional images than good matchers at each level of learning.

To examine how one’s representation changes during face learning and the individual differences in this process, we estimated three parameters in the DPSD (Ro, Rn and Fam) using the frequency of confidence ratings in each phase of the face learning task. We examined individual differences in each parameter and the extent to which exposure to multiple images of the target identity leads to changes in Ro (recollection of the target), changes in Rn (certainty that a face was not previously seen), and/or changes in Fam. We predicted that good vs. poor matchers would differ in how these processes change during learning.

## Data analysis and results

### Data analysis

All analyses were performed using SPSS. We used Bonferroni corrections to control for multiple comparisons in both post-hoc and simple effect analyses. For all tasks we used signal detection theory to measure sensitivity (d′). We arbitrarily defined a hit as responding *same* on match trials (GFMT, AIFMT) or *old* when the target identity was presented in the learning task. Hit rates of 1 were replaced with (*n*_Signal or Noise trials_ − 0.5)/*n* and false alarm rates of 0 were replaced with 0.5/*n*_Signal or Noise trials_^51^.

### Face learning slope

We analyzed whether matching ability predicted face learning in three ways. First, we calculated mean d′ across the two unfamiliar face matching tasks (weighted equally). We used a tertial split on the mean d′ to create three groups that differed in sensitivity (poor matchers, mid-level matchers and good matchers). We then conducted a 3 (matching ability: poor, mid-level, good) × 4 (learning phase: 1, 3, 6, and 9 images) mixed factorial Analysis of Variance (ANOVA) with d′ as the dependent variable. A significant *3 (matching group)* × *4 (learning phase)* interaction would provide evidence that the slope of learning varies with matching ability.

Second, we further probed these findings to investigate whether the slope from one learning phase to another was predicted by average matching d′. To obtain an estimate of the slope, we created residual scores using a regression in which performance in each learning phase was predicted by the preceding phase (e.g., using performance on the 1-image phase to predict performance on the 3-image phase). Measures of slope were obtained for the 3-, 6-, and 9-image phases. These measures of slope were correlated with the continuous measure of average matching ability.

Third, we confirmed our findings by calculating a difference score (9 image d′—1 image d′) for each participant—an analysis that focuses on the start and end point of face learning, consistent with previous research. These results were analyzed using a one-way ANOVA.

### Dual process signal detection

To examine the extent to which individual differences in improvements during face learning reflected changes in familiarity or recollection (old, new), receiver operating characteristics for the learning task were conducted using the DPSD model. Parameters (Ro, Rn, Fam) were estimated using the frequency that participants reported each level of confidence across trial types (target, distractor) and image learning phases using the default settings of the ROC Toolbox in Matlab^52^. Investigations of scatterplots revealed several outliers in the Fam parameter; therefore we used an iterative outlier process (i.e., all participants who scored ± 3 *SD* away from the mean in any condition were removed until there were no more outliers within the data). For consistency we also used this same process for the Ro and Rn parameters. Thus, Fam analyses are conducted with *n* = 94 participants, Ro analyses are conducted with *n* = 129 participants, and Rn analyses are conducted with *n* = 149 participants. Less strict outlier removal strategies yield the same results.

For each parameter we conducted a 3 (matching group; low, mid-level, high) × 4 (learning phase; 1, 3, 6, and 9 images) mixed ANOVA. Significant interactions were followed up with analyses of simple effects in which we analyzed the effect of learning phase at each level of matching ability.

## Results

### Face learning slope

**Figure 2:**
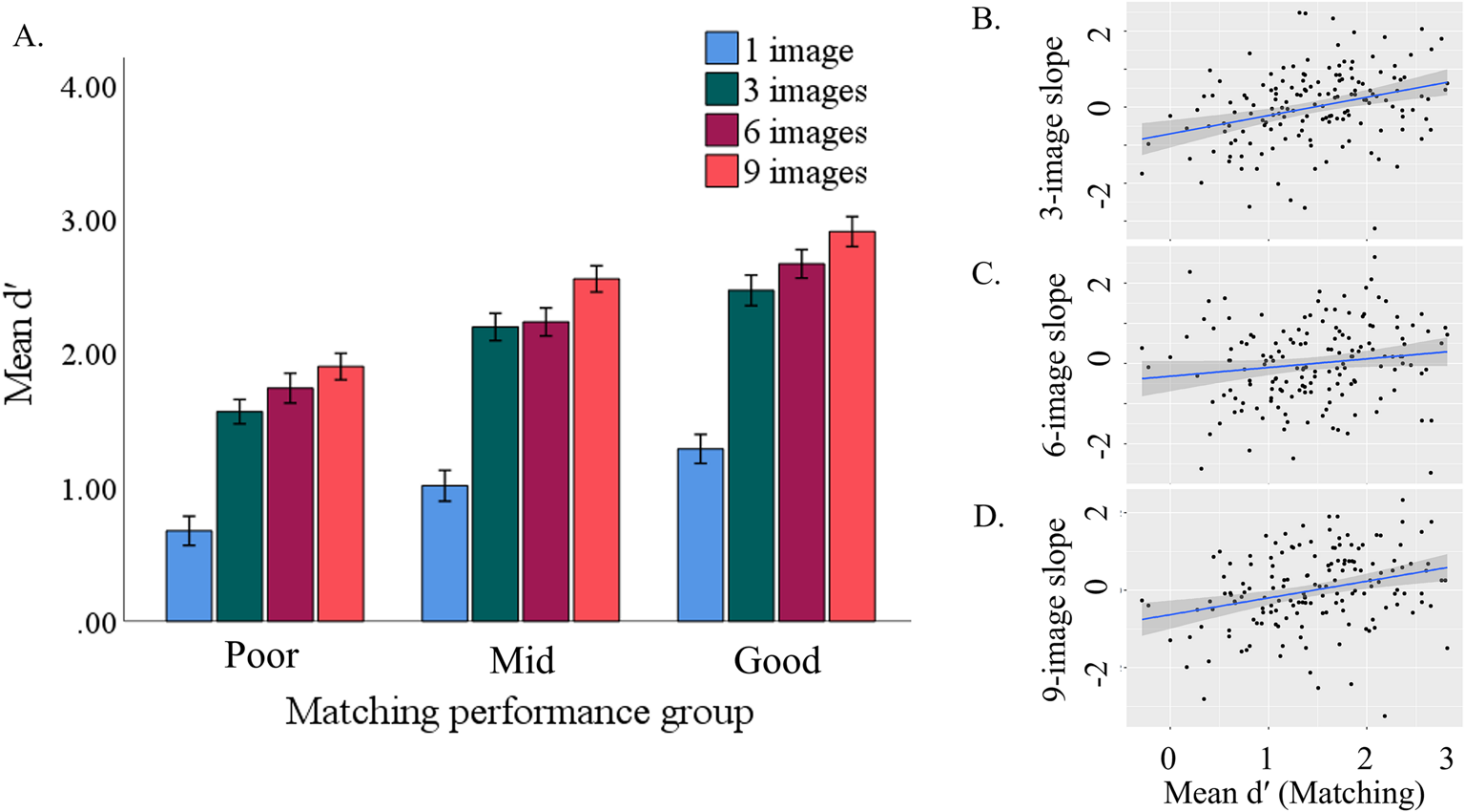
(**A**) depicts mean d′ in each phase of the learning condition as a function of unfamiliar face matching ability. Error bars reflect 95% CIs and are corrected for within-subject error. (**B**)–(**D**) reflect the correlations between average matching ability (d′) and the slope for the 3-, 6- and 9-image phases, respectively.

Matching ability influenced learning across the four phases. The main effects of learning phase (*p* < 0.001, *η*_*p*_^2^ = 0.69) and matching ability (*p* < 0.001, *η*_*p*_^2^ = 0.31) were qualified by a significant interaction, *F*(3,459) = 2.57, *p* = 0.02, *η*_*p*_^*2*^ = 0.03. Simple effects analyses revealed that d′ was higher in the 3-image phase than the 1-image phase (*ps* < 0.001), and the 9-image phase than the 3-image phase (*ps* < 0.002) for all groups, with the larger increase being from 1 to 3 images (see Fig. 2A). d′ was not higher in the 6-image phase than in the 3-image phase for any groups, (*ps* > 0.10) and was higher in the 9- than the 6-image phase for mid-level and good matchers (*ps* < 0.02), but not for poor matchers (*p* = 0.28).

To test whether matching ability predicted the amount of learning at each phase we examined residual scores (See Fig. 2B–D). Matching ability predicted the slope for the 3- (*r* = 0.32, *p* < 0.001) and 9-image phases (*r* = 0.29,* p* < 0.001), such that the benefit of viewing additional images was positively correlated with matching ability. This effect was not observed for the 6-image phase (*r* = 0.14, *p* = 0.07).

The overall slope of learning (from 1 to 9 images) varied between the groups. The ANOVA revealed a main effect of group, *F*(2, 153) = 5.66, *p* = 0.004, *η*_*p*_^2^ = 0.07. Post-hoc analyses revealed that poor matchers showed less improvement (*m* = 1.23, *SD* = 0.62) than both mid-level (*m* = 1.55, *SD* = 1.62) and good matchers (*m* = 1.62, *SD* = 0.65), *p*s < 0.04. Mid-level and good matchers did not differ, *p* > 0.99. Collectively, these findings suggest that poor matchers benefit less from exposure to variability in appearance than do mid-level and good matchers.

### Dual process signal detection

**Figure 3:**
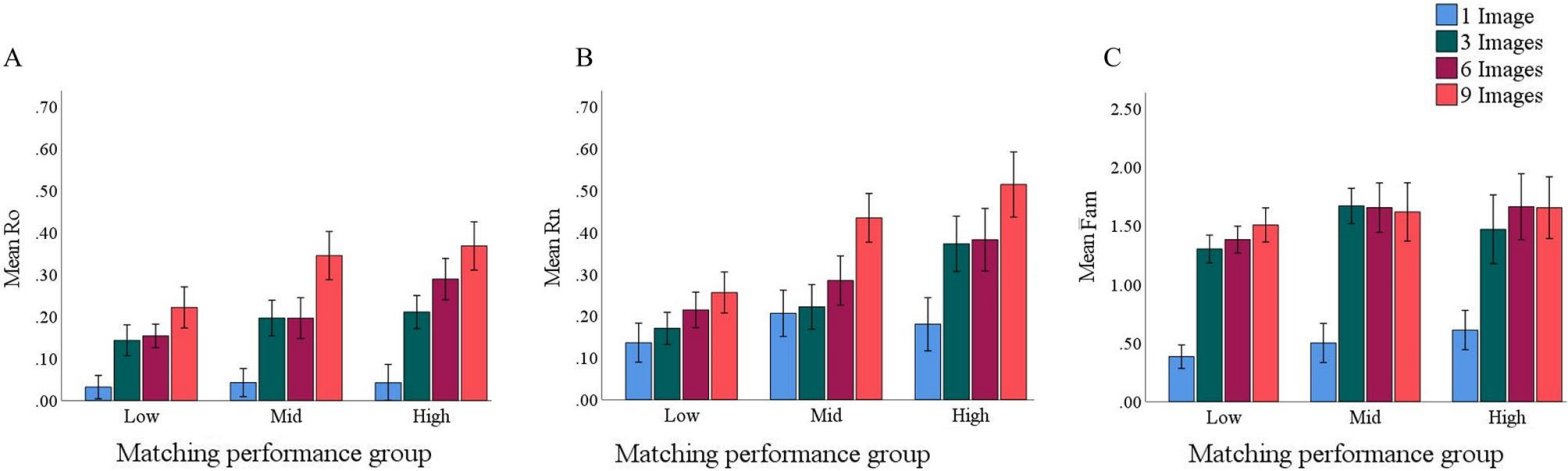
(**A**)–(**C**) depicts mean Ro, Rn and Fam in each phase of the learning condition as a function of unfamiliar face matching ability, respectively. Error bars corrected for within-subject error. Error bars reflect 95% CIs.

**Ro.** The main effect of learning phase for the Ro parameter was significant, *F*(2.51, 328.83) = 61.25, *p* < 0.001, *η*_*p*_^2^ = 0.32. Post-hoc analyses revealed that Ro was smaller in the 1-image phase (*m* = 0.04, *SD* = 0.06) than all other phases, *p*s < 0.001. Ro in the 9-image phase (*m* = 0.31, *SD* = 0.25) was significantly larger than in the 3- (*m* = 0.18, *SD* = 0.19) and 6-image phases (*m* = 0.21, *SD* = 0.22), *p*s < 0.001. No other comparisons differed, *p*s > 0.59. The main effect of matching ability was also significant, *F*(2, 131) = 5.73,* p* = 0.004,* η*_*p*_^2^ = 0.08. Post-hoc analyses revealed Ro was smaller in poor matchers (*m* = 0.14, *SD* = 0.11) than good matchers (*m* = 0.20, *SD* = 0.15), *p* = 0.001. Mid-level matchers (*m* = 0.23, *SD* = 0.13) did not differ from either group, *ps* > 0.09. These effects were not qualified by a significant interaction, *F*(5.02, 328.83) = 2.15, *p* = 0.06, *η*_*p*_^2^ = 0.03, suggesting that changes in high confidence hits did not vary with matching ability. See Fig. 3A.

**Rn.** The main effects of learning phase, *F*(3, 453) = 24.07,* p* < 0.001,* η*_*p*_^*2*^ = 0.14, and matching ability, *F*(2, 153) = 11.41,* p* < 0.001,* η*_*p*_^2^ = 0.13, were qualified by a significant interaction, *F*(6, 459) = 2.55,* p* = 0.02,* η*_*p*_^2^ = 0.03. Simple effects analyses revealed that poor matchers showed no improvement in the Rn parameter across the 1- (*m* = 0.14, *SD* = 0.19), 3- (*m* = 0.17, *SD* = 0.17), 6- (*m* = 0.21, *SD* = 0.23), and 9- image phases (*m* = 0.26, *SD* = 0.25), *p*s > 0.11. Mid-level matchers only showed a significant improvement in Rn in the 9-image phase; their Rn was significantly larger in the 9-image phase (*m* = 0.43; *SD* = 0.31) than in the 1- (*m* = 0.21; *SD* = 0.23), 3- (*m* = 0.22; *SD* = 0.26) and 6-image phases (*m* = 0.28; *SD* = 0.31), *p*s < 0.03. No other differences were significant. Good matchers showed immediate improvement in Rn; their Rn was significantly smaller in the 1-image phase (*m* = 0.18; *SD* = 0.24) than all other phases, *p*s < 0.001. Rn in the 9-image phase (*m* = 0.51; *SD* = 0.34) was significantly larger than in the 3-image phase (*m* = 0.37; *SD* = 0.33), *p* < 0.001. Rn in the 6-image phase (*m* = 0.38; *SD* = 0.34) did not differ from either the 3- or 9-image phases, *ps* > 0.07. Thus, training improved the ability of mid-level and good matchers, but not poor matchers, to perceive a distractor as being new. Mid-level matchers required more exposure to variability than the good matchers to do so. See Fig. 3B.

**Fam.** The main effect of learning phase [*F*(2.68, 257.49) = 77.44, *p* < 0.001,* η*_*p*_^*2*^ = 0.44] was significant. Post-hoc analyses revealed that Fam was smaller in the 1-image phase (*m* = 0.48, *SD* = 0.54) than the 3- (*m* = 1.45, *SD* = 0.73) 6- (*m* = 1.53, *SD* = 0.80) and 9- (*m* = 1.57, *SD* = 0.80) image phase, *p*s < 0.001. No other conditions differed, *p*s > 0.22. Matching ability [*F*(2,96) = 2.20, *p* = 0.12, *η*_*p*_^2^ = 0.04] and the learning phase by matching ability interaction [*F*(5.36, 257.49) = 0.62, *p* = 0.70, *η*_*p*_^2^ = 0.01] failed to reach significance. See Fig. 3C. 

In short, providing perceivers with additional images led to changes in recollection, a pattern that varied as a function of matching ability. Ro varied with matching ability (good > poor), but increased comparably for all groups. Rn also increased with the number of presented images, but only for mid-level and good matchers, with mid-level matchers requiring more variability (9 images) than good matchers (3 images) to show a benefit. Although Fam increased with the number of presented images, it did not vary across groups.

## Discussion

Little is known about the process by which a newly encountered face becomes familiar. Individual differences have tremendous potential for illuminating our understanding of face learning but have been largely neglected^25^. We capitalized on an individual difference approach to address two key questions. (1) Do individual differences in face-matching ability predict the slope of face learning? (2) Are individual differences in learning best captured by recollection or familiarity parameters?

### Do individual differences in matching ability predict the slope of face learning?

Despite evidence suggesting that the slope of face learning might be related to unfamiliar face matching abilities^13,27,28,30–32,35,36^, no studies had directly examined this question. We provide three pieces of evidence that matching ability is related to the slope of face learning. First, whereas all groups were more sensitive to identity after viewing three vs. one image, only mid-level and good matchers were more sensitive to identity (i.e., further refined their representation) after viewing nine vs. six images. Second, individuals with the best matching abilities showed the steepest slopes (calculated using residuals) in the 3- and 9-image phases. Third, poor matchers showed less improvement after viewing nine vs. one image than did mid-level and good matchers. Collectively, these findings suggest that good matchers are better able to capitalize on exposure to within-person variability in appearance when learning a newly encountered face. Whether the patterns we observed persist as familiarity increases (e.g., after seeing a face on multiple days) is a matter for future research.

Our findings inform our understanding of face identification and corresponding models, and suggest that good vs. poor matchers are interacting with faces differently. One possibility is that good and poor matchers attend to different information when identifying faces and/or weigh information differently while making their decision—possibilities that were explored in a recent chapter^53^. For example, facial moles can be diagnostic of identity, and when individuals are instructed to use moles for unfamiliar face identification, their performance improves^54^. Likewise, when novices are trained to use the featural approach used by forensic examiners, their performance improves^55^. These studies suggest that the information to which one attends contributes to unfamiliar face matching—a pattern that our data suggest may extend to face learning.

One published study runs counter to this explanation. That study reported that developmental prosopagnosics and super recognizers are both more sensitive to critical features (features that lead to the perception of a different identity when altered) than non-critical features^56^. However, the stimuli used in that study were tightly controlled images in which one or more features were systematically altered, not ambient images where appearance varies naturally. Future studies could examine whether individual differences in face identification reflect differences in attending to and/or weighting cues using faces that capture idiosyncratic variability in appearance. Future studies could also examine whether attending to these cues improves the slope of face learning. Relating eye movements to performance is a promising avenue to explore this question^57,58^.

Our data are consistent with evidence from Deep Convolutional Neural Networks (DCNNs)—state-of-the-art algorithms informed by the primate visual system^59,60^. A recent study varied both the number of identities and the number of images per identity on which a DCNN was trained^61^. DCNNs that were trained on fewer identities (i.e., had a sparse representation of identity) required more exposure to variability (i.e., more images) to learn new identities than DCNNs that were trained on many different identities. This is consistent with our proposal that a perceiver’s matching ability (a proxy for their representation of unfamiliar faces) constrains the efficiency of face learning.

Our findings also relate to classic models of face perception^62^. Whereas representations of unfamiliar faces are thought to be pictorial and therefor intolerant to variability, representations of familiar faces are robust and tolerant to variability. Poor matchers might struggle to transition from a pictorial to a robust representation for newly learned faces (e.g., they might include pictorial cues within their representation of newly learned identities). The links between individual differences in unfamiliar face matching and the slope of face learning provide a promising avenue for understanding the relationship between, and transition from, unfamiliar to familiar face recognition^25,63–65^. Given the role that variability plays in learning and performance in a host of domains (e.g., language, motor skills), future research should examine the extent to which the pattern we report is domain-general vs. domain-specific^66^.

### Are individual differences in learning best captured by the recollection parameters?

Two processes (recollection and familiarity) influence the recall of an event: Explicit memory details (recollection) and an implicit sense of familiarity^38–41^. No studies had investigated how these parameters change during the process of face learning. Using the DPSD model, we found no evidence that Fam varies across groups that differ in matching ability or that individual differences in Fam during learning covary with matching ability. This is consistent with evidence that there are no age-related changes in Fam despite young adults showing more sensitivity than older adults when tested with newly learned faces^44–47,49^. These results suggest that, although familiarity increases with learning, individual and group differences reflect recollection.

Ro and Rn did change with learning and the pattern of change varied with matching ability. Ro increased as participants learned a newly encountered face, suggesting that recognition was changing in a precise, episodic-like manner for all participants. Ro was higher in good matchers than poor matchers overall, suggesting that better matchers are more likely to recognize individuals in an episodic manner. The increase in Ro across learning phase did not vary across groups, suggesting that exposure to variability in appearance influenced this process similarly across all levels of matching ability. In contrast, Rn increased during learning but only in good and mid-level matchers—the same groups that benefitted from 9 vs. 6 images and who showed more overall improvement during learning. These findings are consistent with recent findings for individual differences in episodic recall and familiarity responses for familiar faces. When asked to recognize actors from *Game of Thrones*, individuals who had better accuracy in the CFMT(a measure of face memory) provided more responses containing accurate semantic information of target identities (comparable to Ro). They also made fewer false alarms and were less likely to rely on feelings of familiarity than poor matchers (i.e., relied on both Ro and Rn)^43^. This suggests that efficient learning reflects both recognition of an identity across variable instances and discrimination of the newly learned face from similar-looking distractors.

Our examination of how face representations change during the process of learning adds to the literature investigating face learning^12,13,15–17,19–21^. Our findings suggest that one’s representation of newly learned identities changes not only at un unconscious level (Fam), but also in a categorical, episodic way (Ro, Rn). This corresponds with previous findings where individuals best recognize actors from instances that they had previously seen^67^ and rate previously seen photos as having a better likeness^68^. It also relates to findings that exposure to idiosyncratic variability facilitates face learning^15,19,20^. As exposure to variability in appearance facilitates face learning, and episodic memories are specific to the individual events (or faces) that created them, it makes sense that representations are being changed in an episodic manner (Ro) allowing confident rejections of similar looking identities (Rn).

Our findings for Ro, Rn and Fam provide insight into group differences. Our results suggest that poor matchers rely less on recollection than good matchers, leading to the prediction that groups that perform worse on unfamiliar face matching tasks might also rely less on recollection when learning a new face than do groups that perform well. For example, older adults might rely more on familiarity than recollection during their recall of faces^49^ because of their relatively poor face matching abilities^47^. Likewise, individuals might rely more on familiarity than recollection when recognizing newly learned other-race faces^48^ because of poor face matching abilities^5,69^. To the extent that a poor ability to match unfamiliar faces is attributable to a less well refined representation of an identity, our data suggest that poor matching ability might impair the ability to build precise, episodic representations—a hypothesis that could be tested with computer models.

On the surface, our data contrast with a recent examination of the effect of familiarity on perceivers’ similarity judgements of within- vs. between-identity face pairs^70^—at least when tested with highly familiar faces. Familiarity increased similarity ratings of within-identity pairs more than it decreased similarity ratings of between-identity pairs—an effect that was most pronounced for super recognizers. Such findings suggest that Ro might play a larger role than Rn during face learning and better differentiate good vs. poor performers. Our data suggest a key role for Rn. The reported increased similarity responses might be partially attributable to all images of a highly familiar face activating the same representation. In classic models of familiar face recognition, Person Identity Nodes (PINs) are theorized to be the backbone of person recognition^62^. PINs comprise identity-specific information, regardless of modality (e.g., an individual’s voice, their face). A person is recognized when the information reaches a threshold. Increased similarity ratings for within-identity pairs might reflect that both images activated the same PIN, emphasizing the role of telling faces together. Future studies could investigate this hypothesis by integrating newly learned faces into White and colleagues’ design and examining the overlap between similarity ratings and confidence judgements.

### Implications for applied settings

Our findings are significant for applied work. There are many applied contexts in which face learning is relied upon, such as missing person searches, eyewitness testimony, and when using CCTV to monitor individuals for illegal activities. Our data suggest that the duration of exposure and the amount of variability to which a perceiver is exposed will have differential benefits for good vs. poor matchers. The processes underlying face identification in applied settings will vary as a function of unfamiliar face matching abilities. Although good and poor matchers might show greater confidence in recognizing the perpetrator of a crime after exposure to variability, only good matchers are likely to show greater confidence for rejecting a similar looking distractor.

Our results also speak to discussions on face training protocols. We showed that Rn (high confidence correct rejections) can improve with exposure to variability—but is dependent on matching ability. Group-level analyses have shown that training improved either performance on match or mismatch trials, but rarely both^10,21,71^. A potential explanation is that only those with high matching abilities show an immediate benefit of variability in Rn and those with poor matching abilities do not get any benefit at all. Using group level statistics and averaging across individual differences may reduce our ability to show an effect of training on d′, at least in good matchers.

## Conclusion

Models of face identification^59,72^ have theorized on the mechanisms underlying face learning and identification. Investigating individual differences can help to further face identification theories^25^. Using an individual difference approach, we showed that (1) individual differences in matching ability predict the slope of face learning, and (2) that individual differences in face learning are best captured by recollection. These findings can inform models of face identification. As we understand little about the transition from, and relationship between unfamiliar and familiar face recognition^25,63–65^, the approach used here provides a fruitful avenue for future research.

## Methods

### Participants

One-hundred fifty-six Caucasian participants (women: *n* = 133; Age: *M* = 21.62, *SD* = 3.79) completed a battery of three face identification tasks. This sample size provides enough power to detect moderate effects. Three additional participants were excluded because of failing attention checks (see below). Participants also completed the CFMT^34^ to identify whether they met the criteria for developmental prosopagnosia. Five participants did so^73^. We retained these participants in our data analysis; the results do not change when they are removed. Just over half (*n* = 84) of the participants were Brock University students who received research credit for their participation. The remaining participants were recruited online via Prolific (www.prolific.co) and were paid £10 for their participation. Each participant gave informed consent. All participants completed the tasks in a fixed order—a key component of individual differences designs^74,75^.

### Stimuli and tasks

Images in the GFMT (half male) were greyscale (1000 × 700 pix), full-faced and shown on a white background^11^. Images within match trials were taken using different cameras. Images in the AIFMT (half male) were colour photographs (652 × 260 pix) shown on a grey background^26^. Images were taken from a variety of sources and included natural variability in appearance. All images in the AIFMT had a roughly frontal view of each model’s face. Images for the learning task (half male) were colour photographs (125 × 167 pix) and were presented with the original background in each image. Learned identities were individuals with whom Canadian participants were expected to be unfamiliar (Fern Sutherland, Philipp Boy, Gigi Ravelli and Donald Stamper); distractor identities for each target were chosen based on physical similarity. Images for the learning task were gathered from Brock University’s Let’s Face It database, a google search and social media (e.g., Instagram, Twitter). The photographs of each model depicted a roughly frontal view of the face. We presented 33 images of each to-be-learned identity (9 training, 20 test, 4 attention checks) and 20 images of each distractor. All images were of White faces.

## Procedure

All procedures received clearance from the Social Sciences Research Ethics Board of Brock University. We carried out the procedures in accordance with the guidelines specified by the Canadian tri-council and the ethics review board at Brock University.

Participants were tested online using Testable (www.testable.org). After providing informed consent, all participants completed three tasks. The order of the tasks and the order in which stimuli were presented within the learning phase of the learning task were both fixed. Doing so ensures that individual differences are not confounded with variance that arises from any effect of task or stimulus order on performance^74,75^.

### GFMT

Participants completed all 40 trials (50% match) of the shortened version of the GFMT^11^. Participants were shown face pairs and asked to indicate whether each pair showed the same person (by pressing “f”) or different people (by pressing “j”). Images remained on screen until participants made a response.

### AIFMT

Participants completed 80 trials (50% match) of the AIFMT^26,50^. Participants were presented with pairs of images and were asked to determine whether the image pair was of the same person or different people. For the purposes of another study, participants indicated their responses on a scale from 1 (certain that the images belong to the same person) to 6 (certain that the images belong to different individuals). In this manuscript selecting 1, 2 or 3 was considered a match response and selecting 4, 5 or 6 was considered a mismatch response. One participant recognized one identity in the AIFMT. This trial was removed from the analyses.

### Face learning task

Participants learned each of four identities, one at a time. Participants’ ability to recognize a learned identity was assessed after viewing 1, 3, 6, and then 9 images; new images were presented during each test phase. Each assessment included five novel images of the to-be-learned identity, five images of a similar looking distractor, and one attention check (see below). Collapsing across all four identities, participants completed 40 test trials (50% target) at each level of training (1, 3, 6, and 9 images). All images in each learning set were presented simultaneously for 20 s and were removed from the screen when the test stimuli were presented. Each test stimulus remained on screen until participants pressed a response key. Participants indicated whether each test image belonged to the target identity using a scale from 1 (certain that the images belong to the target) to 6 (certain that the images do not belong to the target). To create binary responses, we treated 1, 2, or 3 as match responses and 4, 5, or 6 as mismatch responses. The order in which to-be-learned identities and individual learning images were presented was fixed.

### Attention checks

To ensure that participants were attentive during the experiment we included 18 total attention checks. The attention checks in the AIFMT (*n* = 2) comprised match and mismatches. Match attention checks were easily solved because the same image of the target was presented; mismatch attention checks were easily solved because distractors differed from the target identity in age and sex. The attention checks in the learning task (*n* = 16; 4 per identity, with 1 per phase) comprised the first image of each identity on which participants were trained. Participants who failed > 75% of the attention checks were excluded from all analyses (*n* = 3).

## Data Availability

The datasets collected and analysed during the current study are available in the OSF repository, https://osf.io/3crmy/?view_only=056a559ae5f54089821731731c5a7c36.
